# An Unusual Case of Fracture of the Skull—Trephining Recovery

**Published:** 1885-07

**Authors:** W. S. Tremaine

**Affiliations:** Surgeon-in-Chief Buffalo Hospital of the Sisters of Charity, Etc.


### An Unusual Case of Fracture of the Skull—Trephining—Recovery.

By W. S. Tremaine, M. D.,
Surgeon-in-Chief Buffalo Hospital of the Sisters of Charity, Etc.

On December 16, 1884, I was called on by Dr. Banta, of this city, to see with him in consultation, Otto M. German, aged 32, who had been struck, the previous day, on the head by some bricks from a falling house. For a few hours subsequent to the injury, he was conscious and rational.

When I saw him on the 16th instant, about 24 hours after the accident, he was unconscious, without paralysis, with a pulse of 60; respiration slow but not stertorous; temperature normal.

Careful examination of the head showed no external marks of violence. There was some ecchymosis of the left eyelid, with sub-conjunctival effusion, suggesting the possibility of fracture of the base of the skull. As the most careful examination showed no contusion of the scalp, no evidence of depressed or other fracture, so far as manipulation could discover, I recommended that a purgative be given, and further developments awaited. Next day, Dr. Banta informed me that his symptoms were graver, and that the friends were extremely anxious that something positive should be done. It was decided to remove the patient to the Emergency Hospital, where, assisted by Drs. Banta, Mickle and Potter, I again made careful examination, and beyond the very *slightest* ædæma over the left forehead, nothing could be discovered. The man at this time was profoundly unconscious. Pulse, 50; respiration, sighing. I had his head shaved; at about the middle of the upper part of the left parietal bone, a scale of what appeared to be black dirt, about the size of a dime, was scraped off, and beneath it was a faint bruise about half the size of a dime. I made an exploratory semi-lunar incision around this point, turning up the scalp, when I found two fiss ured fractures of the skull passing from behind forward V-shaped. At the apex of the triangle formed by these fissures, a triangular piece of bone was depressed; a linear fissure extended from this forward.

The trephine was applied, and this piece of bone elevated. A clot was found between the cranium and dura mater. On removing this, a rent was discovered in the dura mater, with the clot extending beneath it. A sharp arterial hemorrhage came on, evidently from a vessel in the dura mater. In order to find the bleeding point and secure it, an additional button of bone was removed. About this time the bleeding stopped; it undoubtedly came from a vessel wounded at the time of the accident. The wound was dressed antiseptically with iodoform, after thorough irrigation with mercuric bi-chloride solution, 1 to 1000. Towards the close of the operation, the patient manifested symptoms of returning sensibility; no anesthetic was used. Ten minutes after the last dressing was applied, he gave his name correctly, and asked for something to eat. The next morning consciousness was complete; he was quite rational. Uninterrupted recovery took place; he was discharged, cured, Jan. 12th, 1885.

Of course, the chief interest of this case centers in the difficulty in discovering the exact seat of the lesion in the skull, and demonstrates the utility of thorough search. This man's life was undoubtedly saved by decided and prompt operative interference. It is unnecessary to discuss here the currents and counter currents in regard to the use of the trephine, to be found in surgical literature, the widest difference of opinion prevails. But one thing, to my mind, seems certain, that the operation of trephining adds but little to the danger of the patient, proper antiseptic precautions being taken and competent operative skill understood.

After considerable experience in injuries of this class, and careful reading of the literature pertaining to the subject, I have come to the belief that in cases of doubt the use of the trephine as an *exploratory* measure is justifiable.

The indications for operative interference are admirably set forth in a paper recently read before the American Surgical Association by Dr. Roberts, of Philadelphia.

